# Melanin‐Integrated Structural Color Hybrid Hydrogels for Wound Healing

**DOI:** 10.1002/advs.202300902

**Published:** 2023-05-21

**Authors:** Xinyue Cao, Lingyu Sun, Dongyu Xu, Shuangshuang Miao, Ning Li, Yuanjin Zhao

**Affiliations:** ^1^ Department of Rheumatology and Immunology Nanjing Drum Tower Hospital School of Biological Science and Medical Engineering Southeast University Nanjing 210096 China; ^2^ Oujiang Laboratory (Zhejiang Lab for Regenerative Medicine Vision and Brain Health) Wenzhou Institute University of Chinese Academy of Sciences Wenzhou Zhejiang 325001 China

**Keywords:** drug delivery, hydrogel, melanin, structural color, wound healing

## Abstract

Hydrogel patches have outstanding values in wound treatment; challenges in this field are concentrated on developing functional and intelligent hydrogel patches with new antibacterial strategies for improving healing process. Herein, a novel melanin‐integrated structural color hybrid hydrogel patches for wound healing is presented. Such hybrid hydrogel patches are fabricated by infusing asiatic acid (AA)‐loaded low melting‐point agarose (AG) pregel into the melanin nanoparticles (MNPs)‐integrated fish gelatin inverse opal film. In this system, MNPs not only impart the hybrid hydrogels with properties of photothermal antibacterial and antioxidant, but also improve the visibility of structural colors by providing an inherent dark background. Besides, the photothermal effect of MNPs under near‐infrared irradiation can also trigger liquid transformation of AG component in hybrid patch, resulting in the controllable release of its loaded proangiogenic AA. Attracting, this drug release induced refractive index variations in the patch can be detected as visible structural color shifting, which can be used to monitor their delivery processes. Benefiting from these features, the hybrid hydrogel patches are demonstrated to achieve excellent therapeutic effects for in vivo wound treatment. Thus, it is believed that the proposed melanin‐integrated structural color hybrid hydrogels are valuable as multifunctional patches for clinical applications.

## Graphical abstract

Here, a novel hybrid color‐diffracting hydrogel patch integrated with natural melanin nanoparticles and Asiatic acid is proposed to promote wound healing. The hybrid patches have excellent photothermal antibacterial and antioxidant capacities, as well as enhanced visual distinction during the drug delivery process. The patch exhibits good curative effect on the infected wounds, indicating great potential for wound healing and dynamic monitoring.

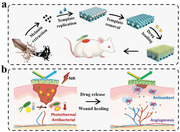

## 1 Introduction

As bacteria invasion would result in severe pain, high lethality, and serious economic burden, the treatment of infected wounds has become a worldwide healthcare problem.^[^ ^1^ ^]^ Given that, a great number of antibacterial drugs have been developed to prevent infection for promoting wound healing, such as antibiotics, herb extracts, bacteriophage, and nanoparticles.^[^ ^2^ ^]^ To implement these drugs to the wound site, diverse drug delivery systems with different forms, including particles, microneedles, and patches, have been explored.^[^ ^3^ ^]^ Among them, wound patches, especially the hydrogel ones, are attractive candidates due to their properties of effective adhesion, high drug‐loading capacity, and flexible applicability.^[^ ^4^ ^]^ Thus, the hydrogel patch‐based drug delivery systems have been applied for treating multifarious skin defects like infected wound, burn wound, chronic wound, etc.^[^ ^5^ ^]^ Although with much progress, most of the existing hydrogel patches are exhibiting simple structures and functions, lacking the capacity of monitoring or even responding to wound states in real time. In addition, the current drugs in treating infection are mostly antibiotics with consequence of ever‐increasing antibiotic resistance, which poses a severe threat to human health. Therefore, it is still anticipated to develop functional and intelligent hydrogel patches with a new antibacterial strategy for wound care.

**Figure 1:**
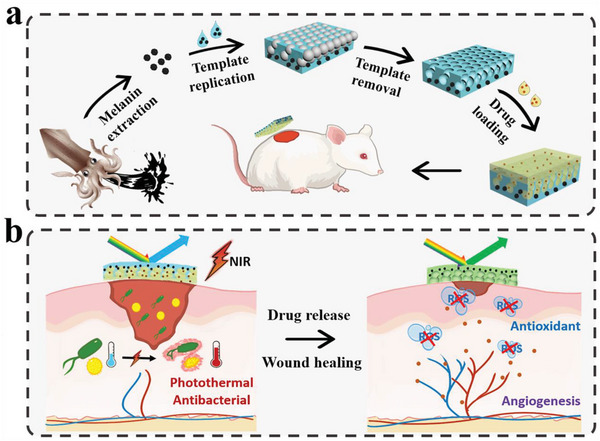
a) Schematic of the preparation progress of the hybrid hydrogel patches. b) Working principle of the hybrid hydrogel patches for promoting bacteria‐infected wound healing.

In this study, we propose a novel melanin‐integrated structural color hybrid hydrogel film with the desired characteristics for wound repair, as schemed in **Figure** **1**. Melanin nanoparticles (MNPs) are a type of natural black biomaterials derived from cuttlefish ink, black hair, or other sources. MNPs exhibit excellent photothermal response properties, intriguing reactive oxygen‐scavenging ability, strong light absorption capacity, as well as inherent biocompatibility and degradability.^[^ ^6^ ^]^ Profiting from these properties, natural MNPs have served as functional additives in a wide range of biomedical applications including antibacterial, photothermal therapy, tissue regeneration, etc.^[^ ^7^ ^]^ By contrast, inverse opal film (IOF) as a polymeric derivative replicated from photonic crystal templates, possesses unique periodic nanopores and visual structural colors.^[^ ^8^ ^]^ By using responsive polymers as the scaffolds of the IOFs or further introducing responsive polymers into the hydrogel IOFs, both of the nanostructures and visual colors of the derived IOFs could generate reversible variation in response to external stimuli.^[^ ^9^ ^]^ These features impart the responsive IOFs with important values in drug delivery, soft robotics, biosensing, healthcare monitoring, and so on.^[^ ^10^ ^]^ Therefore, it could be conceived that the integration of functional black MNPs and IOFs may generate ideal wound patches with improved sensing and therapeutic effect.

Herein, we fabricated the hydrogel patches by using MNPs‐dispersed fish gelatin methacryloyl (FMA) pregel to replicate a colloidal crystal template and infiltrating asiatic acid (AA)‐mixed agarose pregel into the resultant FMA inverse opal scaffold. In this system, the MNPs could not only achieve photothermal antibacterial and antioxidant effects, but also increase the visibility of the structural color by providing a dark background. When irradiated by near‐infrared (NIR) light, the temperature of the hybrid films would increase under the photothermal effect of MNPs, thus causing the liquid transformation of agarose. In this case, the encapsulated AA could be released from the bilayer films, accompanied with variations in structural colors and reflective wavelengths that induced by refractive index changes. Thus, the amount of released drugs could be measured simply by detecting the shifted wavelength values of the hybrid films, providing essential guidance for efficient clinical therapy. In a typical infected wound model, the practicability of the obtained patch has been demonstrated as a useful therapy for wound repair by reducing inflammation, facilitating tissue remodeling, together with serving as an observable sensor for reflecting drug release states. These findings suggested that the multifunctional MNPs‐integrated structural color hybrid hydrogel has practical value as a multifunctional patch for clinical treatment.

## 2 Results and Discussion

**Figure 2:**
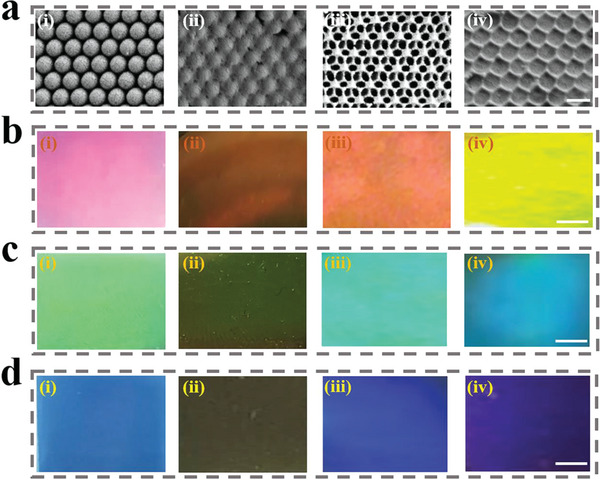
SEM and optical images of hybrid hydrogel films. a) The SEM images and b–d) optical images of the products in the fabrication process. The structural colors of these CCA templates were red in (b), green in (c), and blue in (d). In each panel, (i) is the CCA template, (ii) is the hydrogel penetrated template, (iii) is the IOF layer, and (iv) is the hybrid hydrogel system. The scale bars are 200 nm in (a) and 1 mm in (b), (c), and (d), respectively.

In a typical experiment, we successfully extracted MNPs from the cuttlefish ink sac after multiple steps of centrifugal washing. These MNPs presented a uniform spherical morphology with a diameter of ≈200 nm, as shown in scanning electron microscopy (SEM) images (Figure S1a, Supporting Information). To obtain the desired melanin‐integrated structural color hydrogels, colloidal crystal array (CCA) templates composed of self‐assembled silica nanoparticles were first prepared. The MNPs‐loaded FMA pregel solution was then poured into the CCA template to fully fill its voids, followed by ultraviolet (UV) light irradiation for polymerization. Hydrofluoric acid was finally used to etch the template and the inverse opal hydrogel layer could be obtained. Based on this porous scaffold, the molten agarose (AG) pregel solution was utilized to fill the pores of inverse opal structure. As a common temperature‐sensitive hydrophilic colloid, AG exhibits a low melting point of ≈62 °C. When the temperature dropped to room temperature, the spontaneous gelation of AG could lead to the successful formation of complete hybrid hydrogel system. During the fabrication process, the nanostructures of achieved samples could be observed by SEM. As depicted in **Figure** **2**a, the silica nanoparticles in the CCA templates were packed closely into a hexagonal arrangement. Besides, the resultant inverse opal scaffold displayed periodically interconnected nanopores following the replication of the CCA templates. It could also be seen that the AG hydrogel has fully completely filled the nanopores of the scaffold.

Due to the periodically arranged nanostructures replicated from the CCA templates, this hybrid hydrogel system showed vivid structural color (Figure 2b–d). The interconnected nanopores have imparted the inverse opal scaffolds with photonic bandgap, which could modulate the incident light and only reflect light of specific wavelengths. Notably, brilliant structural color could be observed when the light's wavelength was within the range of visible light. In general, Bragg's equation could be used to predict the value of the reflection wavelength (*λ*)

(1) $$\mathrm{Bragg}{}^{\prime }s\;\mathrm{equation}:\;\lambda =1.633dn_{\mathrm{average}}$$

In Bragg's equation, *d* stands for the distance between the centers of neighboring nanoholes while *n*_average_ represents the hydrogel materials’ average refractive index. According to this equation, the reflection wavelength might alter if *n*_average_ varies. Given that, when AG hydrogel filled the nanopores of the inverse opal scaffold, the corresponding change of hybrid hydrogel's refractive index would further lead to the change of reflection wavelength value, together with the visual structural color of the hydrogel film. Herein, to analyze the regular change pattern in visible structural colors, various IOFs with distinct structural colors were generated. Compared with IOFs, the visual structural colors of the resultant hybrid hydrogel films generally generated an obvious blue‐shift (Figure 2b–d). It is notable that such refractive index‐induced structural color change has been proved to be reversible, attributed to the phase‐change capacity of AG (Figure S1c, Supporting Information). Specifically, when temperature increased, the AG hydrogel would transform to liquid status and then release from the IOFs, causing the recovery of visual structural color. In this regard, this refractive index‐induced structural color change could serve as a visual sensor, thus providing more potential for biomedical applications.

**Figure 3:**
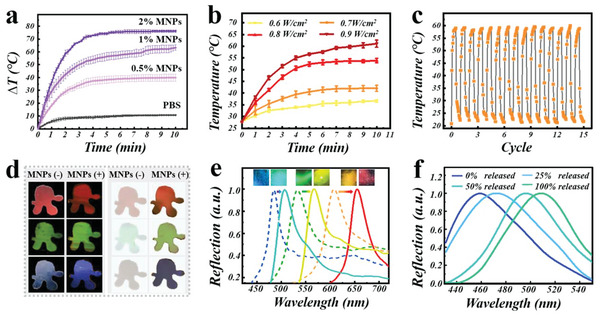
The photothermal and drug release properties of the MNPs‐integrated hybrid films. a) Temperature changes of MNPs solution at different concentrations with NIR irradiation (808 nm, 1 Wcm^−2^, 10 min). b) Temperature changes of MNPs‐doped hybrid hydrogel film with NIR irradiation at different intensities (808 nm, 10 min). c) The temperature changes of MNPs‐doped hybrid hydrogel film during 15 laser on/off cycles. d) The optical images of common IOF (MNPs (−)) and MNPs‐doped IOF (MNPs (+)) on black and white backgrounds, respectively. e) The structural color and the reflection peak change during the AG release process. Dashed lines represent the reflection peak before the AG release, and solid lines represent the reflection peak after the AG release. f) The red‐shift reflection peaks of hybrid film during drug release process.

Different from other photothermal conversion elements, such as black phosphorus, carbon nanotubes, and graphene oxide, MNPs are natural components possessing inherent properties of biocompatibility, degradability, chelating capability, and intriguing free radical scavenging ability. Especially, with excellent light‐absorption efficiency, MNPs could exhibit great photothermal performance with a desirable conversion efficiency. Their photothermal transduction behavior was then assessed by using an NIR laser (808 nm, 1 Wcm^−2^, 10 min) to irradiate MNPs solutions at different concentrations. For the PBS solution without MNPs, its temperature increased by only 10 °C. While MNPs solutions presented a clear concentration‐dependent heating characteristic from 40 °C at 0.5% (w/v) to 75 °C at 2% (w/v), suggesting their favorable photothermal transduction properties (**Figure** **3**a). Furthermore, MNPs showed great photothermal conversion stability during the five on/off irradiation cycles (Figure S2, Supporting Information). Considering the high photothermal conversion efficiency, 2% MNPs were introduced into the FMA inverse opal scaffold, as observed by SEM (Figure S1b, Supporting Information). The added MNPs could impart the patches with similar photothermal properties, whose temperature changes under NIR irradiation (808 nm, 0.6–0.9 Wcm^−2^) for 10 min were also analyzed. In view of the actual application on the wound, the NIR irradiation power of 0.9 W cm^−2^ was chosen. In this case, the patches’ temperature rapidly heated up and reached a plateau at about 55 °C after 4 min, and then gradually increased to about 60 °C (Figure 3b). Similar to pure MNPs, these MNPs‐doped films presented great photothermal conversion stability in fifteen on/off irradiation cycles as well, as shown in Figure 3c.

In addition to the photothermal conversion efficiency, the added MNPs could also increase the contrast of the structural color as a dark background. After the MNPs were introduced to the FMA pregel solution to form a homogeneously dispersed solution, this mixture solution was utilized to fill the voids of the CCA templates. As the diameter of MNPs was ≈200 nm, only FMA pregel solution could infiltrate into the voids of the CCA templates. As a result, the final IOFs were composed of the dark hydrogel part (MNPs‐doped FMA hydrogel) and the inverse opal scaffold part (FMA hydrogel only). In comparison with common IOFs made of FMA, both of them could be well visualized and easily detected by spectrometers over a black background. However, the structural color of the common transparent IOFs was difficult to be observed or detected on a light color background, especially a white one (Figure 3d). This phenomenon was ascribed to the apparent light transmission and reduced reflection that caused by the comparatively low refractive index contrast. By contrast, the inherent black view of MNPs‐doped IOFs contributed to the color contrast improvement, so that the clearer structural color could be observed even on the white background. This background‐independent capacity would greatly improve its detection convenience and widen its application in biosensing area.

Benefiting from the high specific surface area derived from porous structure, the IOFs also gained brilliant drug‐carrying capacity. Especially, accompanying with stimuli‐responsive components, IOFs could further possess adjustable interconnected nanostructures and structural colors, which are raising increasing interest and research on developing intelligent drug delivery systems.^[^ ^11^ ^]^ Herein, the added MNPs could impart the hybrid hydrogel system with intelligent NIR‐responsive properties. In this system, AA as the model drug was encapsulated in AG hydrogel and subsequently loaded into IOFs, with the aim of promoting the growth of new blood vessels for wound repair.^[^ ^12^ ^]^ Without NIR irradiation, AA was gradually released from the AG, with a release rate of ≈75% after 24 h (Figure S3a, Supporting Information). By contrast, upon NIR irradiation, the temperature of MNPs‐integrated IOFs would increase and further melt the reversible phase‐changing AG hydrogel, causing the release of encapsulated AA from the scaffold at the same time. During this drug release process, the visual structural color and reflection peak of patches exhibited red shifting, as shown in Figure 3e. To quantify this process, the relationship between the AG release amount and the reflective wavelength shift value was analyzed. As displayed in Figure 3f, the reflection peak of the patch presented a persistent red shift along with phase transformation and drug release process, until it approached the wavelength of the original inverse opal scaffold. Figure S3b of the Supporting Information additionally demonstrated the possibility of a linear relationship between the drug release percentage and the red‐shift value, indicating that these IOFs can be useful for self‐monitoring during the drug release procedure.

**Figure 4:**
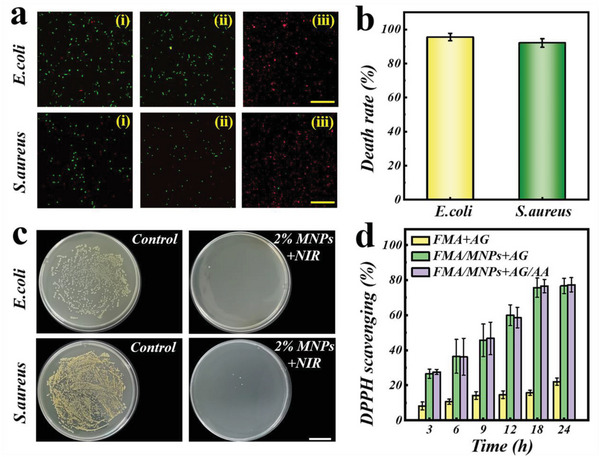
In vitro antibacterial and antioxidant experiment. a) Live/Dead staining of *E. coli* and *S. aureus* treated with (i) PBS, (ii) MNPs‐doped hybrid hydrogel films without NIR irradiation, and (iii) MNPs‐doped hybrid hydrogel films with NIR irradiation (808 nm, 0.9 Wcm^−2^, 15 min). The live and dead bacteria were stained by SYTO (green) and PI (red), respectively. b) Bacterial death rate of *E. coli* and *S. aureus* treated with MNPs‐doped hybrid hydrogel films with NIR irradiation. c) Photographs of *E. coli* and *S. aureus* colonies with PBS, and MNPs‐doped bilayer hydrogel films with NIR irradiation. d) The scavenging effect on DPPH of different groups. Scale bars are 5 µm in (a) and 2 cm in (c).

Subsequently, we examined the hybrid patches’ photothermal antimicrobial activity based on their photothermal properties. The results of bacterial living and death staining indicated that both the *Escherichia coli* and *Staphylococcus aureus* almost survived in PBS buffer and unirradiated hybrid films‐contained PBS buffer without NIR irradiation. However, after the hybrid hydrogel films in bacterial suspensions was subject to 15 min of NIR irradiation (0.9 Wcm^−2^), the bacterial suspensions’ temperature remained at 61 °C, leading to bacterial death. According to the photothermal antibacterial results (**Figure** **4**a,b), the MNPs‐loaded hybrid films showed an antibacterial efficiency of ≈95% of *E. coli* and 92% of *S. aureus*, respectively. Their antimicrobial capacities were additionally confirmed using an agar plate dilution method. Compared with the negative control, nearly none colony could be found in the experimental group (Figure 4c). In addition to antibacterial activity, we verified the reactive oxygen species (ROS)‐scavenging capacity of hybrid films as the integrated MNPs possess numerous antioxidant groups such as phenolic hydroxyl. The results of in vitro antioxidant test were displayed in Figure 4d and Figure S4 (Supporting Information), where all MNPs‐loaded hybrid films showed higher 1′‐diphenyl‐2‐picrylhydrazyl radicals (DPPH) scavenging capacities. Notably, the films’ scavenging capacity against DPPH demonstrated a clear MNPs concentration‐dependent manner. Especially, when the concentration of MNPs reached 20 mg mL^−1^, the scavenging effect on DPPH was ≈80%. Taking advantages of these excellent photothermal antibacterial and antioxidant activities, the MNP‐loaded hybrid films would be beneficial to maintain a sterile environment and scavenge harmful ROS at the wound area to accelerate the wound healing procedure.

Next, we evaluated the hybrid films’ biocompatibility and hemocompatibility. When incubated with hybrid films, NIH‐3T3 cells exhibited normal cell proliferation behavior. The cell staining results also revealed that the NIH‐3T3 cells had satisfying morphology and survivability (Figure S5, Supporting Information). Besides, a hemolysis test was further carried out to evaluate the hybrid films’ blood‐compatibility. Notably, the results revealed that IOFs barely generated any hemolysis when in contact with red blood cells (Figure S6, Supporting Information). Since AA has a certified effect in promoting angiogenesis and facilitating relevant growth factor genes expression in fibroblast, it was encapsulated into the composite hydrogel system to enhance the clinical value of the hybrid patches.^[^ ^12^ ^]^ To certify this, the in vitro tube formation experiment was first carried out. With the sustaining release of AA into the cell culture medium under NIR irradiation, the cyclization of vascular cells had been greatly enhanced in the AA‐contained medium. Compared with other groups, this bioactive drug‐contained group achieved desired angiogenesis effect (Figure S7, Supporting Information). These results suggested that these hybrid films were safe materials with the potential to facilitate wound healing.

**Figure 5:**
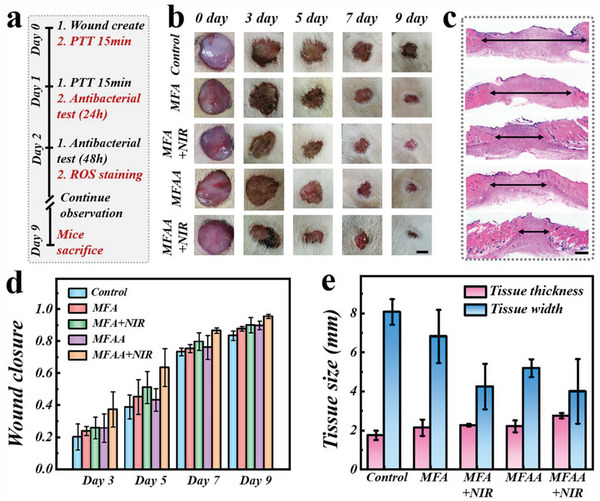
Wound closure process and HE staining. a) Time schedule of bacteria‐infected wound model. b) Representative photographs of the skin wounds in control group, MFA group, MFA + NIR group, MFAA group, and MFAA + NIR group. c) HE staining of wounds after 9 days. d) The statistical result of the wound closure situation. e) Quantitative analysis of granulation tissue width and thickness. Scale bars are 5 mm in (b) and 1 mm in (c).

To further assess the practical therapeutic value of this hybrid film in vivo, infected full‐thickness skin defect animal models were conducted. As schemed in **Figure** **5**a, in this experiment, skin area with a diameter of 1.5 cm was removed away from the Sprague Dawley (SD) rat back, followed by the injection of mixed bacterial solution into wound sites to form infection. SD rats were randomly separated into five groups, each comprising three rats, including the MNPs@FMA + AG group (MFA group), MNPs@FMA + AG with NIR irradiation group (MFA + NIR group), MNPs@FMA + AA@AG group (MFAA group), MNPs@FMA + AA@AG with NIR irradiation group (MFAA + NIR group) and PBS group (control group). The wound site statuses during the wound repair processes had been recorded on days 0, 3, 5, 7, and 9 for the next elaborate assays. Photos in Figure 5b clearly suggested that the wounds in patch‐treated groups had a higher closure rate and narrower granulation tissue width compared to the control group, since FMA itself had already been proved a collagen biomaterial with the potential to provide an extracellular matrix‐mimicking microenvironment for wound healing. According to quantitative analysis of these photos, it could be intuitively seen that the MNPs‐integrated groups under NIR irradiation had relatively sped up the wound healing process (Figure 5d).

Besides, to show the new granulation tissues in the wound regions more accurately, hematoxylin–eosin (HE) staining of the wound sites was carried out after 9 days. As displayed in the optical images and the quantitative analysis of the HE staining results (Figure 5c,e), the MFAA + NIR group not only demonstrated the smallest granulation tissue width (2.17 mm), but also owned the thickest granulation tissue of 2.83 mm. More importantly, photothermal therapy (PTT) was carried out to achieve in vivo antibacterial and drug release effect after the infected wounds were created. The agar plate dilution method was employed to confirm the in vivo antimicrobial activity caused by PTT. It was clearly found that the PTT treated group had less bacterial colony formation than the control group, especially after 48 h (Figure S8, Supporting Information). Furthermore, we tested the antioxidant activity of the MNPs and AA doped bilayer patches through ROS staining (Figure S9, Supporting Information). Compared with the control group and FMA group, an obvious decrease in the fluorescent intensity could be seen in the MFA group and MFAA group, indicating the effective in vivo antioxidant properties of hybrid patches.

**Figure 6:**
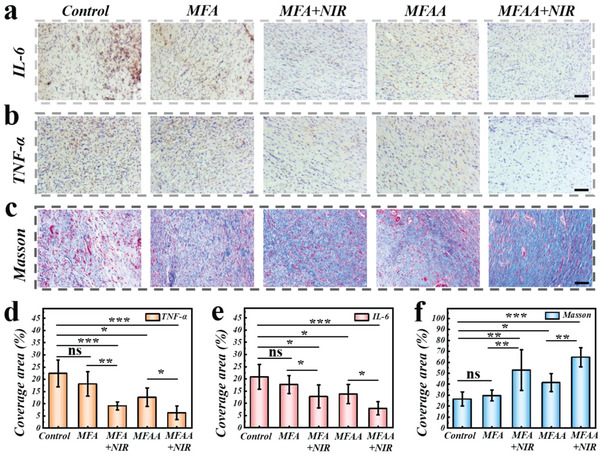
Immunostaining images of a) IL‐6, and b) TNF‐*α* in different groups. c) Masson's trichrome staining images in different groups. d–f) Statistical analysis of positive coverage area of (d) IL‐6, (e) TNF‐*α*, and (f) collagen deposition. ns, not significant, * *p* < 0.05, ** *p* < 0.01, *** *p* < 0.001. Scale bars are all 200 µm.

There are relatively different phases during wound healing process, while inflammation phase mainly involves several phagocytosis events to clear out damaged components, such as dead cells, pathogens, and debris, and reduce infections. During the inflammation phase, multiple inflammatory factors are expressed to help immune cells to proliferate and differentiate.^[^ ^13^ ^]^ The inflammatory factors’ expression amounts are frequently used to indicate the severity of wound infection. In this study, immunohistochemical staining has been utilized to assess the expression amounts of interleukin‐6 (IL‐6) and tumor necrosis factor‐*α* (TNF‐*α*), which were considered as two representative inflammatory cytokines. Because of the severe inflammatory reaction, the staining results revealed the highest expression of inflammatory factors in the control group (**Figure** **6**a,b). Meanwhile, the least inflammatory factors’ amount was detected in the MFAA + NIR group, which mainly benefited from the antibacterial effects of PTT and AA. These results may be more intuitively expressed by the statistical data on the positive area coverage of these two inflammatory factors (Figure 6d,e).

Remodeling, the last phase of the healing process, is defined by the transition from granulation tissue to wound closure. Since the status of collagen deposition is often implemented to evaluate the wound remodeling level, such indicator was assessed by using Masson's trichrome staining in this study. The results indicated that the patch‐treated groups showed enhanced collagen deposition (Figure 6c). As expected, the MFAA + NIR group owned the best collagen deposition mainly, due to the bioclean wound environment caused by the photothermal antibacterial effect. Notably, revascularization is another typical indicator to assess the tissues’ remodeling condition. Given that, immunostaining of CD31 was applied to evaluate the angiogenesis condition in wound region (Figure S10, Supporting Information). In the control group, only a tiny number of novel created blood vessels could be found, which was might owing to the high inflammatory level's inhibition of cell proliferation and differentiation. By contrast, an obviously higher new blood vessels’ density was found in the MFAA + NIR group, profiting from the accelerating angiogenesis effect of AA. To sum up, due to the combined therapy based on MNPs and AA, the statistical analysis also showed that the MFAA + NIR group was superior to the other two experimental groups in infection inhibition, collagen deposition, angiogenesis, and tissue regeneration (Figure 6f; Figure S10, Supporting Information). These consequences suggested that this melanin‐integrated hybrid patch could be applicable in the wound treatment field.

## 3 Conclusion

To sum up, we have proposed a novel hybrid structural color hydrogel patch integrated with natural MNPs and AA to promote wound healing. This patch was fabricated by infusing AA‐loaded low melting‐point AG pregel into the MNPs‐doped FMA hydrogel inverse opal scaffolds. The added MNPs had not only imparted the hybrid patches with capacities of photothermal antibacterial and antioxidant, but also successfully increased the contrast of the structural color as a dark background. Furthermore, benefiting from the phase‐changing property of AG, the encapsulated AA could sustainingly release from the hybrid hydrogel to the wound site during the photothermal therapy process. This drug release has further induced refractive index and visual structural color changing, which could be applied to monitor its delivery processes. When applied for in vivo wound healing studies, the wound healing and remodeling have been greatly accelerated due to the therapeutic effect of MNPs and AA. These characteristics suggested that this multifunctional hybrid film would have promising applications as an infected‐wound therapeutic method.

## 4 Experimental Section

### Materials and Animals

Cuttlefish ink sacs were purchased from local market (Nanjing, China). Low melting‐point AG and AA were purchased from Aladdin. FMA, and CCA templates were all made in Zhao's laboratory.^[^ ^14^ ^]^ Twenty SD rats (gender: male; age: 8–12 weeks; weight: 200 g) were prepared for this study. Notably, all the animal experiments were permitted by the Animal Investigation Ethics Committee of Nanjing Drum Tower Hospital (Approval No.: 2021AE02013).

### MNPs Extraction

Using a simple differential centrifugation technique, the MNPs were separated from the fresh ink sac. The MNPs solution was first centrifuged at 1000 rpm (10 min) to remove big particles, and then at 10 000 rpm (10 min for five times) to extract MNPs. The final MNPs were suspended in deionized (DI) water after being cleaned numerous times with DI water.

### Fabrication of Hybrid Hydrogel

A vertical deposition method was first used to prepare a series of CCA templates with different silica nanoparticle diameters (230, 260, and 320 nm). As for the MNPs@FMA inverse opal scaffold, FMA (150 mg) and HMPP (10 µL) were added in DI water (1 mL) to disperse into a homogeneous solution, which was then mixed with MNPs solution (100 µL, 20 mg mL^−1^) to obtain the pregel solution. This composite solution was poured into the CCA templates and solidified upon UV irradiation. Finally, the IOFs were obtained after soaking in 4% hydrofluoric acid to etch the templates. As for the filler layer, 2% AG solution was prepared. After heating, the molten AG pregel solution was injected into the IOFs. The AG pregel would solidify when temperature dropped to room temperature. Therefore, the final hybrid hydrogel film was obtained.

### In Vitro Drug Release Experiment

The AA's concentration was 1 mg mL^−1^ in 2% AG pregel solution. Then the drug‐loaded AG pregel was dropped into the IOF and solidified spontaneously. Then the hybrid hydrogel film was placed in the PBS solution and irradiated by NIR (808 nm, 0.9 W cm^−2^). The hybrid hydrogel films’ spectra were also detected with increasing temperature and liquid conversion of AG.

### In Vitro Photothermal Antimicrobial Test

For the purpose of assessing the hybrid films’ photothermal antimicrobial activity, the 2% MNPs‐integrated hybrid patches with 1 cm diameter were first made. The bacteria (*E. coli* and *S. aureus*) were then resuspended in sterile PBS with a final turbidity of 0.5 MCF. Next, each hole in the 48‐well plate received 500 µL of bacterial solution and two patch pieces, with PBS addition serving as the control group. After the patch‐involved solution was irradiated under NIR (808 nm, 0.9 W cm^−2^, 15 min), SYTO and PI were employed to carry out Live/Dead staining (SYTO 30 min, PI 2 min). Finally, the bacterial survival states were demonstrated using a fluorescent microscope. Additionally, the standard plate with solid medium was then added along with the diluted bacterial suspension. After incubating the bacteria at 37 °C overnight, the bacterial colonies were counted.

### In Vitro Antioxidant Test

DPPH was first dissolved in ethylalcohol solution with the origin OD value of 0.8–1 measured by a microplate reader. Samples were all cut into 1 cm diameter and located in holes with DPPH solution (2 mL). While a hole containing only DPPH solution (2 mL) served as the control group. In an orbital shaker, they were then incubated at 37 °C. Every 2 h, the samples’ absorbance (Abs) at 517 nm was recorded. Each sample's antioxidant activity was estimated using this equation

(2) $$\mathrm{Antioxidant}\;\mathrm{activity}\left(\%\right)=\frac{{\mathrm{Abs}}_{\mathrm{control}}-{\mathrm{Abs}}_{\mathrm{sample}}}{{\mathrm{Abs}}_{\mathrm{control}}}\times 100\%$$

### In Vitro Biocompatibility Experiment

MTT assessment was carried out. In 48‐well plates, the NIH‐3T3 cells were separated into four groups (Figure S5, Supporting Information). Followed with the procedure of MTT assessment, the cells’ relative activities were finally measured. The living cells were further stained with calcein‐AM. Finally, a fluorescence microscope recorded the staining results of the cells on day 3.

### In Vitro Tube Formation Experiment

The DMEM medium was supplemented with 15% FBS and 1% double antibiotics. HUVECs were grown in this medium at 37 °C temperature and 5% CO_2_. A 48‐well plate's bottoms were first paved with Matrigel, which was then solidified for 20 min in the cell incubator. Then a new medium was used to cultivate 3 × 10^4^ mL^−1^ HUVECs. Next, three different media were employed to cultivate cells: medium (control group), medium after soaked with MNPs@FMA + AG film (MFA group), and medium after soaked with MNPs@FMA + AA@AG patch (MFAA group). Calcein‐AM staining was used to show tubes’ production status after 6 h of incubation. Additionally, ImageJ program was used for the statistics analysis.

### In Vivo Skin Tissue Regeneration of Infected Wound

For the purpose of evaluating the therapeutic value of hybrid hydrogel films, bacterial‐infected full‐thickness skin defect models were created. The SD rats were sedated before having their back fur and skin removed (1.5 cm diameter). The wounds’ surfaces were then injected 200 µL of a mixed bacterial suspension (*E. coli* and *S. aureus*). SD rats were randomly separated into five groups (each comprising four rats), including the including PBS group, MFA group, MFA + NIR group, MFAA group, and MFAA + NIR group, conducted different treatments, as schemed in Figure 5a. After adding the hydrogel film at the wound area, NIR irradiation was used to implement in vivo photothermal antibacterial therapy. Its power was adjusted to bring the temperature of the hydrogel film up to 60 °C. Notably, direct NIR irradiation to the mouse skin should be prevented during the treatment. Besides, for the in vivo antioxidant test, the wounds were collected on day 2 and underwent ROS fluorescent staining. Next, a digital camera was used to record the wounds status on day 0, 3, 5, 7, and 9. Besides, these regrowing tissues were collected after 9 days. These tissues were then embedded in paraffin and cut into 5 µm slices for subsequent staining. The width and thickness of these regrowing tissues were then shown using HE staining. To gauge the level of inflammation, immunohistochemical staining of TNF‐*α* and IL‐6 was also done. To show the collagen deposition, Masson's trichrome staining was utilized. Finally, immunohistochemical staining of CD31 was carried out to demonstrate the status of new blood vessel creation.

### Characterization

Using a scanning electron microscope (SEM, Hitachi S‐3000N), the MNPs’ morphology and the hybrid films’ nanostructures were examined. An optical microscope (Olympus, BX51) fitted with a fiber‐optic (Ocean Optics, USB2000‐FLG) spectrometer was used to detect reflection spectra. The optical microscope was used to study the stained sample pieces (OLYMPUS BX51). ImageJ software was used to implement quantitative analysis of photographs.

### Statistical Analysis

For each data, at least three independent experiments were conducted. The obtained data were expressed as mean ± SD. SPSS R26 was applied for statistical significance analyses. The significant difference between different groups was analyzed with one‐way ANOVA with significance as follows: ***p < 0.001, 0.001 < **p < 0.01, 0.01 < *p < 0.05, as *p* > 0.05 was considered to have no significant differences (ns).

## Conflict of Interest

The authors declare no conflict of interest.

## Author Contributions

Y.Z. conceived the idea. X.C. conducted experiments and data analysis. X.C. wrote the manuscript. L.S., D.X., S.M., and N.L. assisted with paper writing.

## Supporting information

Supporting Information

## Data Availability

The data that support the findings of this study are available in the supplementary material of this article.
