# Supplementary material for: Melanin‐Integrated Structural Color Hybrid Hydrogels for Wound Healing
Source: Adv Sci (Weinh). 2023 May 21;10(22):2300902. doi: 10.1002/advs.202300902 (PMC10401079; doi:10.1002/advs.202300902)
Supplement: Supplementary file 1 — Supporting Information [file ADVS-10-2300902-s001.pdf]

ADVANCED SCIENCE
Open Access

Supporting Information

for *Adv. Sci.*, DOI 10.1002/advs.202300902

Melanin-Integrated Structural Color Hybrid Hydrogels for Wound Healing

*Xinyue Cao, Lingyu Sun, Dongyu Xu, Shuangshuang Miao, Ning Li and Yuanjin Zhao**

Supplementary

Melanin-integrated structural color hybrid hydrogels for wound healing

Xinyue Cao[1], Lingyu Sun[1], Dongyu Xu[1], Shuangshuang Miao[1], Ning Li[1], Yuanjin Zhao[1,2]*

[1]Department of Rheumatology and Immunology, Nanjing Drum Tower Hospital, School of Biological Science and Medical Engineering, Southeast University, Nanjing 210096, China.

[2]Oujiang Laboratory (Zhejiang Lab for Regenerative Medicine, Vision and Brain Health), Wenzhou Institute, University of Chinese Academy of Sciences, Wenzhou, Zhejiang 325001, China.

†Corresponding author. Email: yjzhao@seu.edu.cn

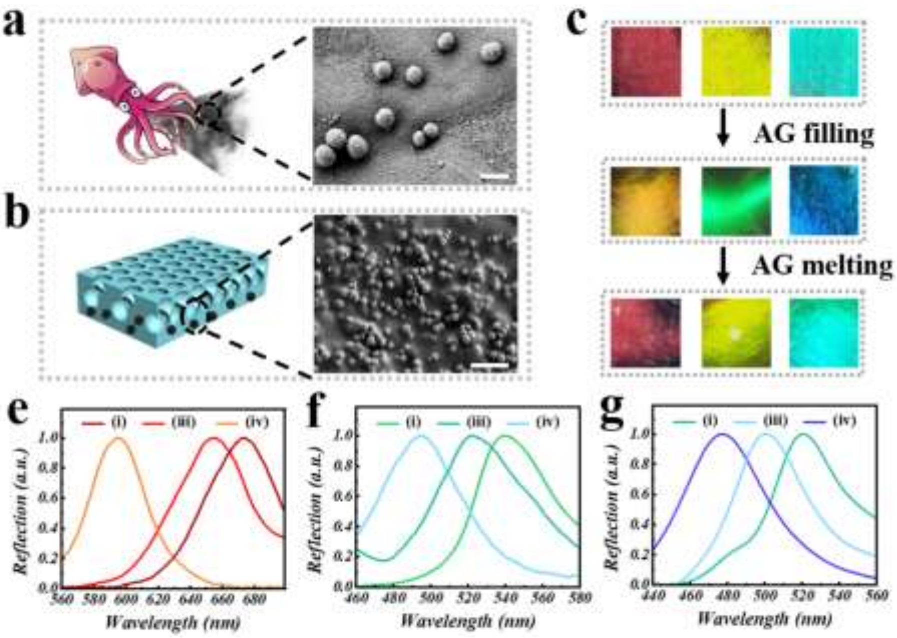

Figure S1. The morphology of the MNPs-integrated hybrid films. (a, b) The schematic diagram and SEM image showing MNPs in the ink sac of cuttlefish (a) and

MNPs-doped IOFs in (b). Scale bars are 200 nm in (a) and 1 μm in (b). (c) The visual color change accompanying with the different states of AG gel. (e-f) The reflection peak change during the hybrid hydrogel fabrication process. (i) is the CCA template, (iii) is the IOF layer, and (iv) is the hybrid hydrogel system.

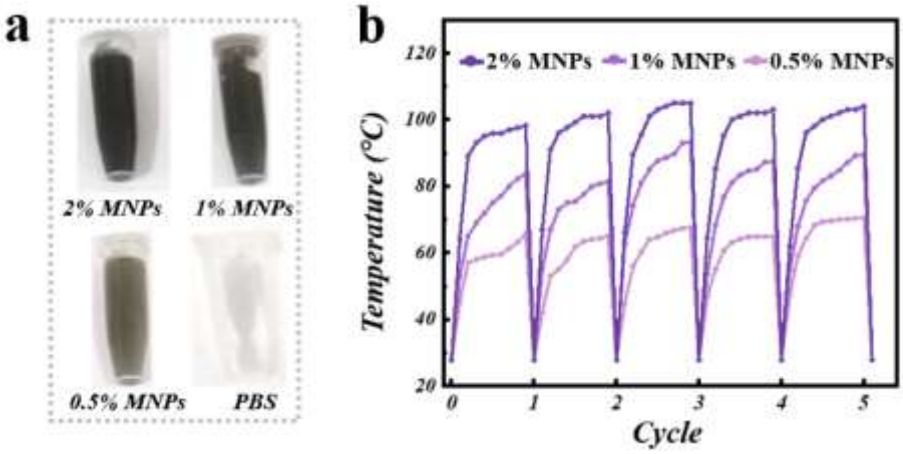

Figure S2. The images and photothermal properties of the MNP solutions. (a) Photographs of MNPs solutions with different concentrations. (b) The temperature change of MNPs solution with different concentration during five laser on/off cycles (808 nm, 1 W cm^{-2}).

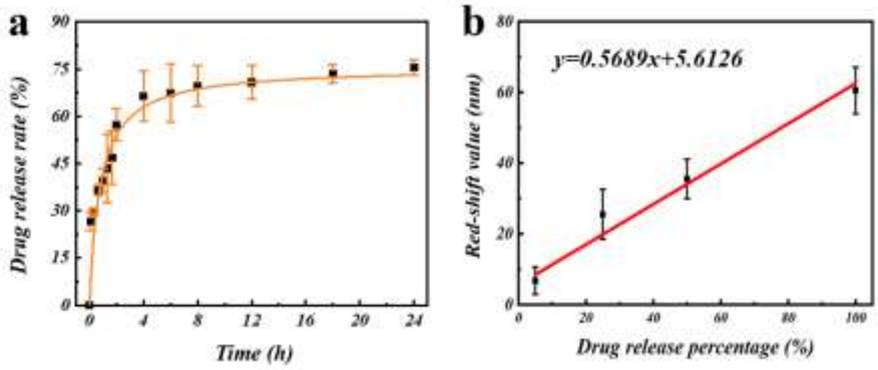

Figure S3. (a) The drug release rate curve without NIR irradiation. (b) The relationship between red-shift value and drug release percentage.

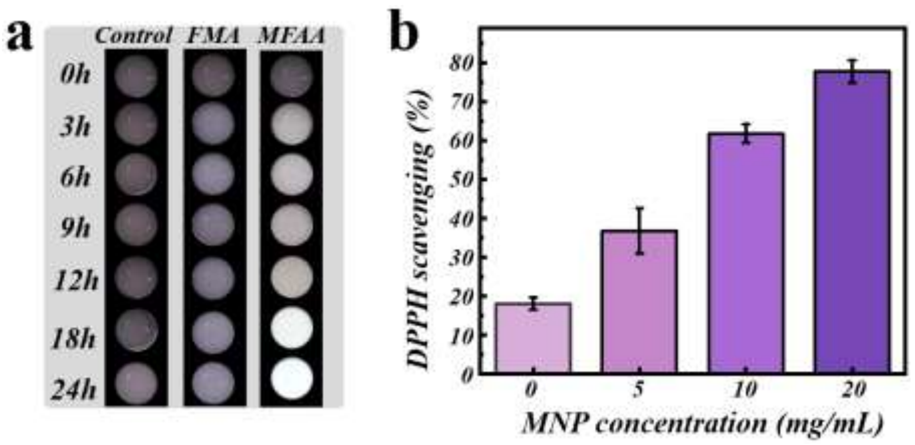

Figure S4. DPPH scavenging test. (a) The images of DPPH scavenging process in different groups. (b) The MNPs concentration-dependent DPPH scavenging activity.

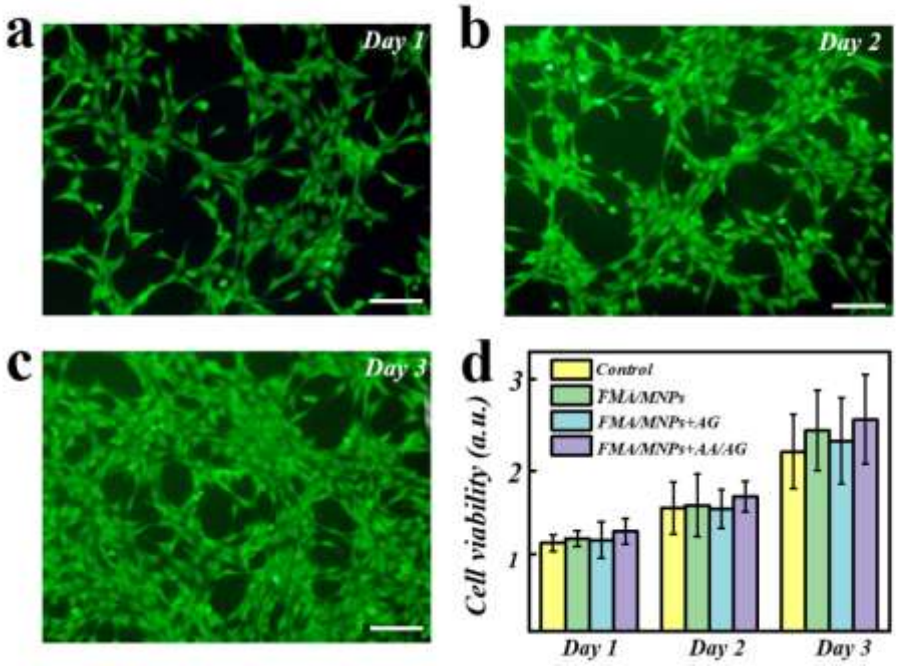

Figure S5. Investigation of the biocompatibility ability. Fluorescence images of cells cultured with the hybrid films on day 1 (a), day 2 (b), and day 3 (c). (d) Statistical analysis of the cell viability in different groups. The scale bars are 50 μm.

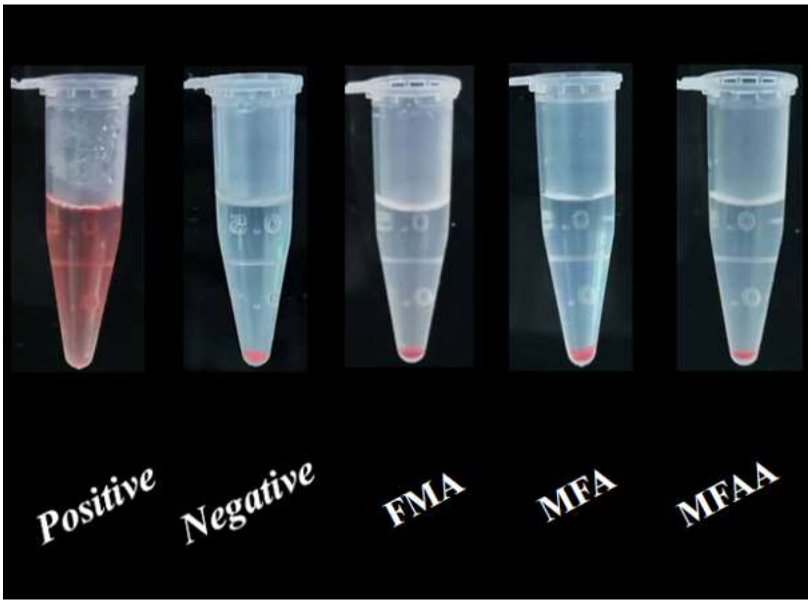

Figure S6. The photographs of hemolysis test results.

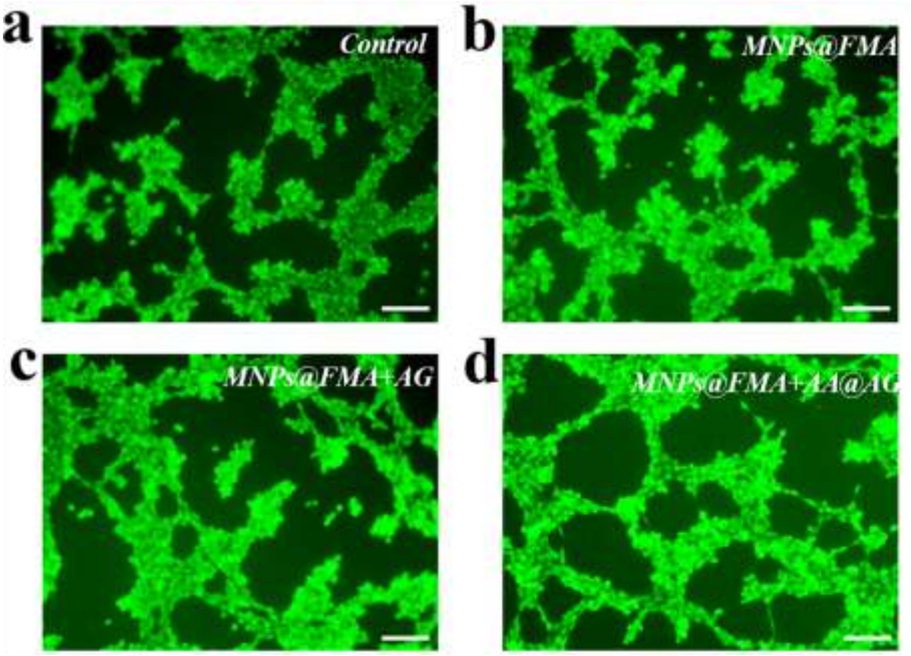

Figure S7. Fluorescent images of tube formation in different groups. The scale bars are 50 μm.

μm.

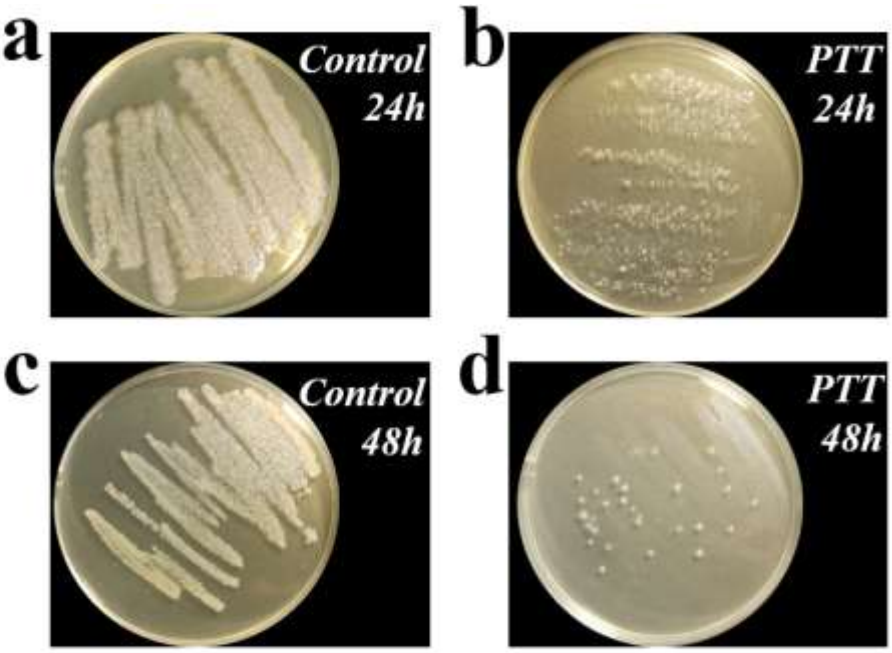

Figure S8. *In vivo* antibacterial test. (a, b) Photographs of *E. coli* and *S. aureus* colonies with PBS, and MNPs-doped hybrid hydrogel with NIR irradiation on day 1. (c, d) Photographs of *E. coli* and *S. aureus* colonies with PBS, and MNPs-doped hybrid hydrogel with NIR irradiation on day 2.

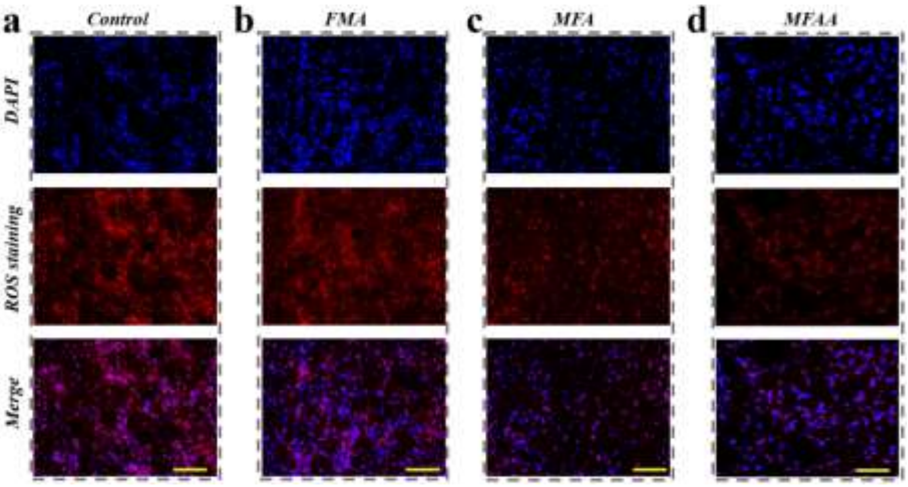

Figure S9. (a-d) ROS fluorescence staining in different groups. Scale bars are 200 μm.

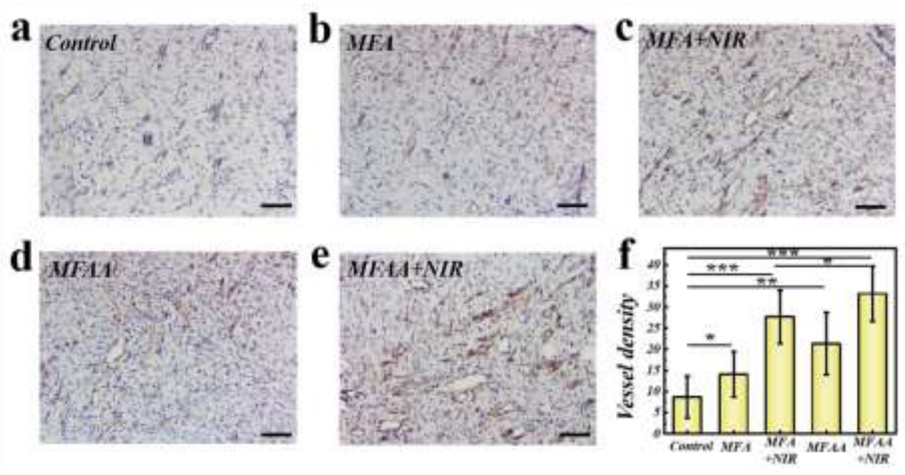

Figure S10. (a-e) Immunostaining of CD31 in different groups. (f) Statistical analysis of the total vessel density. Scale bars are 200 μm. * $p < 0.05$, ** $p < 0.01$, *** $p < 0.001$.
